# Massive edema of ovary with cytogenetic alteration of chromosome 12q13-15 in adolescent patient: a case report

**DOI:** 10.1186/1757-2215-6-13

**Published:** 2013-02-07

**Authors:** Rossella De Cecio, Monica Cantile, Nicola Fortunato, Annarosaria De Chiara, Nunzia Simona Losito, Renato Franco, Gerardo Botti

**Affiliations:** 1 Pathology Unit, Istituto Nazionale Tumori Fondazione “G. Pascale”, via Mariano Semmola, 80131, Napoli, Italy; 2 Pathology Unit, National Cancer Institute, Pascale Hospital, via Mariano Semmola, 80131, Naples, Italy

**Keywords:** Ovarian edema, Sarcoma-like, CHOP translocation

## Abstract

**Background:**

The massive edema of ovary, with or without fibromatosis, is a rare tumor-like entity characterized by an increase in volume of one or both ovaries for accumulation of edema fluid in the stroma that separates the follicular structures.

**Case:**

We report a rare case, very peculiar also for its association with a massive stromal fibromatosis and for the presence, never described, of tumoral areas with CHOP gene translocation, on chromosome 12q13–15.

## Background

The massive ovarian edema is a rare benign lesions mimicking neoplastic lesions and typically affects young women more frequently in the second and third decades of life.

Edema involves one or both ovaries and its etiology is related to partial and intermittent ovarian pedicle torsion that interfere with lymphatic drainage. The edema typically presents with abdominal pain less frequently with menstrual disorders [1].

Androgenic manifestations (virilization, hirsutism ect.) are described among the symptoms, due to the presence of luteinizzate stromal cells that are found in 30/40% of cases [2]. An abdominal mass ranging in size from 5 cm to about 35 cm can be appreciated on palpation.

## Case presentation

A 15-year-old girl has been admitted to gynecological surgery of our Institute for the presence of suspect ovarian masses. During surgery, frozen sections excluded malignancy.

**Figure 1:**
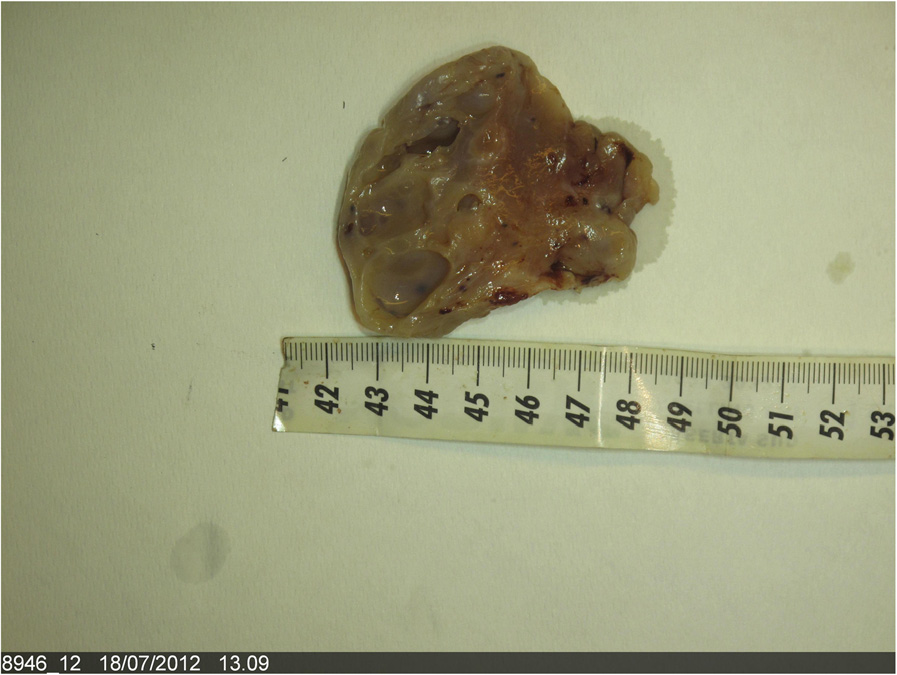
Ovarian mass with compact solid areas including jelly-like cysts (cut section).

Grossly the annessectomy showed an ovarian mass (Figure 1) of 6.5 × 4 × 3 cm dimension, with small, pink and translucent at cut surface reliefs, with compact solid areas including jelly-like cysts. In addition, a nodular formation in the left paraovaric region, of 5 × 4 × 3 cm dimension was present, with reliefs similar to that observed in the ovary.

**Figure 2:**
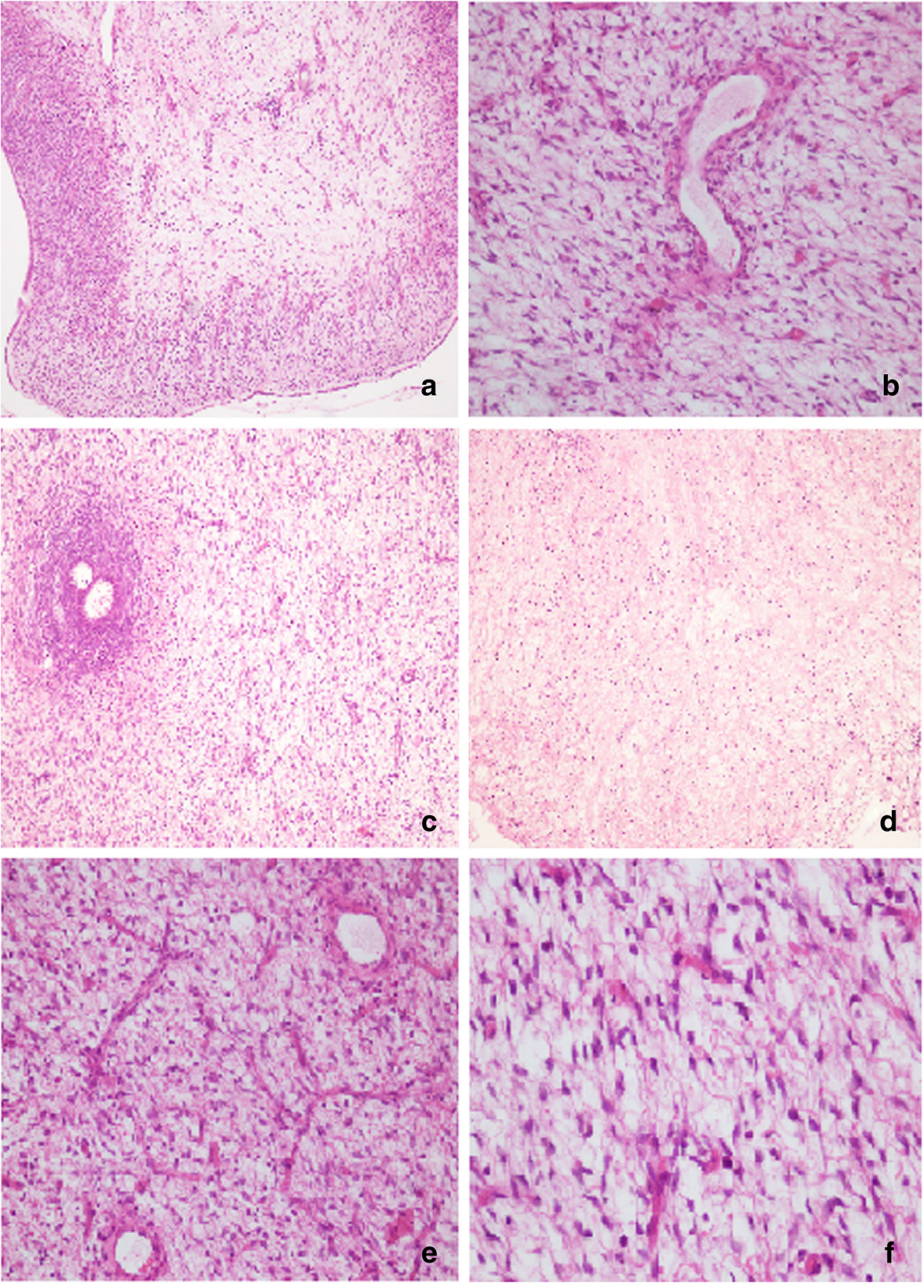
H & E: a) spindle cells with myofibroblastic and storiform pattern (10X); b) follicular cysts in cortex area (10X); c) edematose stroma around secondary follicles (10X); d) foci of edema (10X), e) sarcoma-like area (20X); f) sarcoma-like area (40X).

Microscopically, both lesions were characterized by a diffuse proliferation of spindle cells with myofibroblastic and storiform pattern, separated by thick bands of collagen, trapping inclusion and follicular cysts, especially in the cortex (Figure 2). In addition, interspersed areas with loose and edematose stroma had been observed around secondary follicles (Figure 2). The massive edema was mainly present in medullary region, while it was absent in the cortex. In the oedematous areas stellate cells have been found with scanty, interposed collagen, associated to sinusoids-like vessels (sarcoma-like) (Figure 2). Mitoses were generally absent, but numerous in some areas. Furthermore, foci of extravasation and congestion were widely observed.

**Figure 3:**
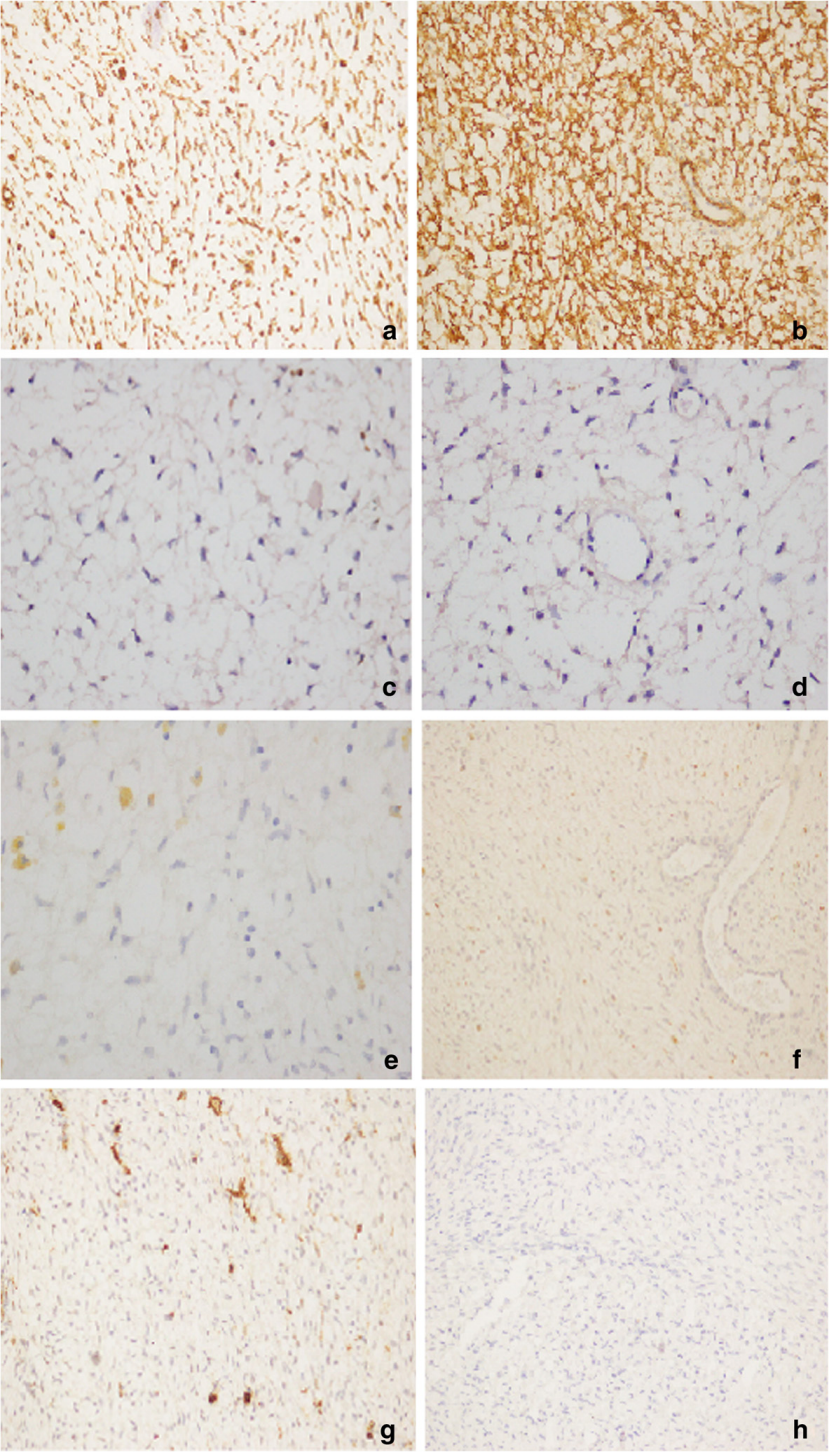
Myofibroblastic cells: a) positive immunostaining for Vimentin (20X); b) positive immunostaining for Actin (20X); c) negative immunostaining for S −100 (20X); d) negative immunostaining for Ki 67 20x; e) ) negative immunostaining for Inhibin (20X); f) negative immunostaining for Desmin (20X); g) ) negative immunostaining for CD34 (20X); h) ) negative immunostaining for Calretinin (20X).

Immunohistochemical study showed cells positivity for vimentin and muscle specific actin and negativity for alpha-inhibin, desmin, S-100, calretinin and CD34 (Figure 3).

**Figure 4:**
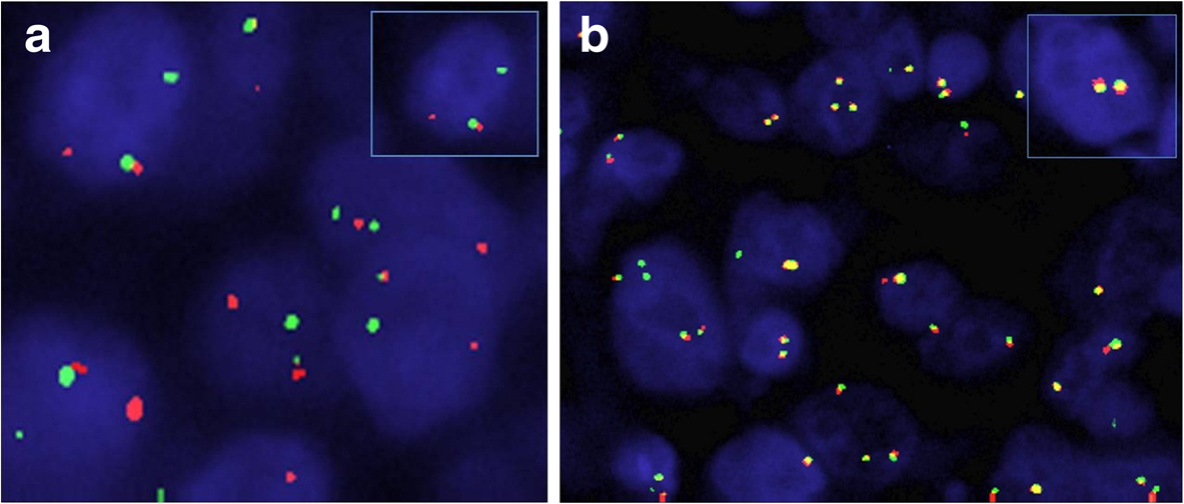
Representative samples of break-apart FISH assay for CHOP rearrangement: a) area with the presence of the intact CHOP gene; b) area representative of CHOP disruption. Arrows mark the cells of interest, which are enlarged in the insets.

Moreover, a cytogenetic investigation was also performed through FISH using a commercial probe (Vysis® LSI *CHOP* Dual Color Breakapart Probe, Abbott Molecular) to verify the presence of aberrations related to the 12q13-15 chromosomal region. The FISH analysis revealed heterogeneous areas; particularly oedematus areas with stellate elements showed the presence of breakage of the CHOP gene. Overall, the percentage of translocated tumor cells is 10% in whole section, while in areas with more consistent chromosomal aberrations the percentage of cells with breakage of CHOP gene is approximately 80% (Figure 4).

The sample had been submitted to RT-PCR analysis, after RNA purification, to verify the presence of the fusion transcripts CHOP/FUS and CHOP/EWS [3,4], typically encountered of myxoid liposarcoma. However, the molecular analysis did not reveal the presence of these transcripts.

## Conclusions

In case that we have described, in addition to massive edema, the presence of a significant stromal fibromatosis was observed. Both aspects seem to be the final event of the same disorder (torsion of the pedicle and primitive stromal proliferation).

Moreover, in fibromatosis area follicles are surrounded by stromal myofibroblastic proliferation [1]. Before the confirmation of this diagnosis, it is important exclude other ovarian diseases. The differential diagnosis should be made with sclerosing stromal tumor, the thecoma luteinized with sclerosing peritonitis and the ovarian myxoma [5].

The sclerosing stromal tumor has a characteristic pseudo-lobular pattern with focal hyalinization and compression of the surrounding ovarian tissue. Differently from edema and fibromatosis this cancer does not spare the follicles, the tumor involves only one ovary and the age of onset is most frequently around 27 years. The immunohistochemical profile is characterized by a positivity of stromal cells for Vimentin, calretinin, alpha-inhibin and CD34.

The myxoma is a benign tumor of the ovary characterized by a myxoid stroma in which stellate cells and a vascular component consists of small capillaries are immersed; the age most frequently affected is between 16 and 45 years and is usually unilateral.

The cells are positive for Vimentin and muscle specific actin.

The thecoma luteinized with sclerosing peritonitis consists of spindle cells mixed with luteinized cells with diffuse edema with many mitoses and in 30% of cases occur in women under 30 years. The neoplastic cells were positive for alpha-inhibin and calretinin. These lesions have in common the change of follicles that are not recognizable.

The morphological analysis and immunohistochemical staining of our case has confirmed the diagnosis of massive edema of the ovary with fibromatosis, however, the sample also showed suspicious myxoid-like areas. For this reason, we decided to realize a cytogenetic analysis typical of myxoid liposarcoma to detect the CHOP gene translocation.

In the literature only one case of ovarian myxoid liposarcoma with this chromosomal aberration in a young woman has been described [6]. However, the presentation of the lesion is extremely heterogeneous and being present areas with translocated cells. Furthermore, the analysis of RT-PCR performed to identify the fusion transcripts CHOP/FUS and CHOP/EWS [7,8] was negative. Therefore, we exclude that this is a rare case of myxoid liposarcoma of ovary.

Our data can suggest the existence of a different fusion partner for CHOP, but, obviously, more detailed molecular studies are required to support our hypothesis.

For the pathologist and the clinician is important to know this benign disease that for its presentation may mimic a neoplastic pathology. In fact, the massive edema of ovary generally occurs in young women where the reproductive capacity must be preserved, avoiding surgery demolition. Moreover, the development of a more adequate molecular characterization, is necessary not only for the differential diagnosis of this malignance.

### Consent

Written informed consent was obtained from the patient for publication of this Case Report and any accompanying images. A copy of the written consent is available for review by the Editor-in-Chief of this journal.

## Competing interests

The authors declare that they have no competing interests.

## Authors’ contributions

DC, ADC, RF, NSL examined macroscopically and microscopically surgical specimen and performed the immunohistochemical staining and FISH analysis of the tumor. MC, NF and GB conceived of the study, and helped to draft the manuscript. All authors read and approved the final manuscript.
